# The impact of multimodality integrated positron emission tomography-computed tomography on improving the staging and management of head and neck malignancy: a cross- sectional study

**DOI:** 10.1590/1516-3180.2021.0599.R1.15092021

**Published:** 2022-04-02

**Authors:** Sethu Thakachy Subha, Abdul Jalil Nordin

**Affiliations:** I MS. Associate Professor, Department of Otorhinolaryngology, Faculty of Medicine and Health Sciences, Universiti Putra Malaysia (UPM), Serdang, Selangor, Malaysia.; II MD. Professor, Department of Imaging, Nuclear Diagnostic Imaging Centre, Faculty of Medicine and Health Sciences, Universiti Putra Malaysia (UPM), Serdang, Selangor, Malaysia.

**Keywords:** Head and neck neoplasms, Nasopharyngeal carcinoma, Neoplasm staging, Positron emission tomography computed tomography, American Joint Committee on Cancer, Contrast enhanced computed tomography, Head and neck malignancy, Nasopharyngeal cancer, TNM staging

## Abstract

**BACKGROUND:**

Clinical assessment of head and neck cancers is highly challenging owing to the complexity of regional anatomy and wide range of lesions. The diagnostic evaluation includes detailed physical examination, biopsy and imaging modalities for disease extent and staging. Appropriate imaging is done to enable determination of precise tumor extent and involvement of lymph nodes, and detection of distant metastases and second primary tumors.

**OBJECTIVE:**

To evaluate the initial staging discrepancy between conventional contrasted computed tomography (CT) and 18F-fluorodeoxy-D-glucose positron emission tomography/computed tomography (^18^F-FDG PET/CT) and its impact on management plans for head and neck malignancies.

**DESIGN AND SETTING:**

Prospective cross-sectional study in two tertiary-level hospitals.

**METHODS:**

This study included 30 patients with primary head and neck malignant tumors who underwent contrasted computed tomography and whole-body ^18^F-FDG PET/CT assessments. The staging and treatment plans were compared with the incremental information obtained after ^18^F-FDG PET/CT.

**RESULTS:**

^18^F-FDG PET/CT was found to raise the stage in 33.3% of the cases and the treatment intent was altered in 43.3% of them, while there was no management change in the remaining 56.7%. ^18^F-FDG PET/CT had higher sensitivity (96% versus 89.2%) and accuracy (93% versus 86.7%) than conventional contrast-enhanced computed tomography.

**CONCLUSION:**

Our study demonstrated that ^18^F-FDG PET/CT had higher sensitivity and accuracy for detecting head and neck malignancy, in comparison with conventional contrast-enhanced computed tomography. ^18^F-FDG PET/CT improved the initial staging and substantially impacted the management strategy for head and neck malignancies.

## INTRODUCTION

Head and neck cancers are the sixth most common type of cancer worldwide and the majority of them cause regional nodal metastases that decrease the chances of survival.^1,2^ Head and neck cancer is characterized by high prevalence of nodal metastases at the time of initial presentation.^1-3^ A large percentage of these cause regional nodal metastases that decrease the chances of survival by 50%.^3^ Accurate timely staging will ensure proper treatment delivery.^4,5^ Computed tomography (CT) and magnetic resonance imaging (MRI) are the standard imaging modalities used for the staging evaluation of head and neck cancer in routine clinical practice.^5^ However, the limitations of these morphological imaging methods include difficulty in differentiating reactive enlargement and tumor-infiltrated lymph nodes and difficulty in detecting unsuspected distant metastases.^6^

## OBJECTIVE

This study was conducted to evaluate the role of 18F-fluorodeoxy-D-glucose positron emission tomography/computed tomography (^18^F-FDG-PET/CT), in comparison with contrast-enhanced computed tomography (CECT) for management of patients with head and neck cancer.

## METHODS

This was a prospective cross-sectional study involving 30 patients who were attended at the otorhinolaryngology clinics of two tertiary-level hospitals in Malaysia after obtaining institutional ethical approval (NMRR-09-1116-4585; dated July 13, 2010). Informed consent was obtained from all the enrolled patients.

The exclusion criteria were that the subjects should not be children, individuals with acute or chronic inflammatory disease, pregnant patients, lactating mothers, terminally ill patients or patients with any previous malignancy. All the patients selected (above 18 years old) were thoroughly examined by otorhinolaryngology surgeons, and biopsies were taken from suspicious regions.

All the patients underwent CECT and whole body ^18^F-FDG PET/CT examinations at the hospital’s center for diagnostic nuclear imaging, using a standard protocol for image acquisition. Staging of the disease was done based on the 7^th^ edition of the tumor, node and metastasis (TNM) staging system of the American Joint Committee on Cancer (AJCC) after use of both imaging modalities. In addition, the oncologist was asked to outline the management intent for the patients, based on CECT; and to do this again after the positron emission tomography/computed tomography (PET/CT).

The change in management intent and the incremental information obtained after both imaging procedures had been done were compared and analyzed. The percentage of management changes implemented due to discrepancies between the imaging methods was recorded. The clinical impact of PET/CT was considered ‘high’ if it changed the treatment modality, and ‘low’ if there was no change in the treatment modality or intent.^7^

All the patients were monitored through regular follow-up at a specialist clinic. The cumulative survival rate among the patients was estimated from the date of diagnosis to the date of death due to any cause or the date of the last follow-up. It was noted whether any patients were lost to follow-up or were still alive at the end of the follow-up period. The five-year overall mean survival rate was calculated, and the mean survival time in months according to sociodemographic characteristics, tumor stages and treatment received was also analyzed.

## RESULTS

Out of the 30 patients in this study, 60% (18/30) were male and 40% (12/30) were female. According to ethnicity, the majority were Chinese (56.7%; n = 17), followed by Malay (40%; n = 12) and Indian (3.3%; n = 1). The mean age (with standard deviation, SD) was 49.9 years (± 14.5) with first and second peak age incidences in the age ranges of 30-39 years and 60-69 years, respectively. Nasopharyngeal carcinoma was the commonest malignancy (56.7%), and the next commonest was carcinoma of the larynx and malignancy of the oropharynx (10%). A list of the primary sites of tumors is shown in Table 1, while the patients’ characteristics, clinical and histopathological diagnosis are presented in Table 2.

**Table 1.** List of primary sites of tumors

Primary site	Number of cases	Percentage
Nasopharyngeal carcinoma	17	56.6
Carcinoma of thyroid	2	6.7
Carcinoma of larynx	2	6.7
Carcinoma of unknown primary origin (CUP)	1	3.3
Lymphoma	1	3.3
Carcinoma of hard palate	1	3.3
Carcinoma of base of skull	1	3.3
Carcinoma of tonsils	1	3.3
Sarcoma of tonsils	1	3.3
Adenocarcinoma of base of skull	1	3.3
Others	2	6.7

**Table 2.** Patient characteristics and clinical and histopathological diagnoses

Patient No.	Gender	Age	Clinical diagnosis/stage	Histopathological diagnosis	Follow-up
1	M	63	Thyroglossal cyst	Papillary carcinoma of thyroid	Alive
2	M	44	NPC/stage IV B	NPC	Lost
3	M	60	Carcinoma larynx/stage I	Carcinoma of larynx	Alive
4	F	60	Lymphoma of parotid	Oncocytoma of parotid	Alive
5	M	38	Lymphoma of tonsils	Sarcoma of tonsils	Alive
6	F	31	Benign cyst	Papillary carcinoma of thyroid	Alive
7	M	44	Benign nodule	Occult neck node carcinoma TxN1M0	Alive
8	F	73	Adnexal tumor/stage I	Adnexal carcinoma	Alive
9	F	62	Carcinoma of larynx/stage I	Carcinoma of larynx	Alive
10	F	56	NPC/stage III	NPC	Dead
11	M	50	NPC/stage II	NPC	Dead
12	M	70	NPC/stage I	NPC	Dead
13	M	39	NPC/stage III	NPC	Alive
14	M	37	Carcinoma of tonsils/stage IV B	Carcinoma of tonsils	Alive
15	M	63	Lymphoma/stage I	Lymphoma	Dead
16	M	22	NPC/stage II	NPC	Alive
17	F	68	Mastoiditis	Metastatic adenocarcinoma	Dead
18	F	63	Carcinoma of oropharynx/stage II	Carcinoma of oropharynx	Dead
19	M	65	NPC/stage III	NPC	Dead
20	M	46	NPC/stage III	NPC	Dead
21	M	42	NPC/stage IV B	NPC	Alive
22	F	55	NPC/stage III	NPC	Dead
23	M	54	NPC/stage III	NPC	Dead
24	F	33	NPC/stage II	NPC	Alive
25	M	39	NPC/stage II	NPC	Alive
26	M	41	NPC/stage III	NPC	Alive
27	F	33	NPC/stage II	NPC	Lost
28	F	34	NPC/stage IV B	NPC	Lost
29	M	50	Carcinoma of larynx T2N0M0/stage II	Carcinoma of larynx	Alive
30	F	26	NPC/stage III	NPC	Lost

NPC = nasopharyngeal carcinoma; CT = computed tomography; PET/CT = positron emission tomography/computed tomography; M = male; F = female.

All of the 30 patients underwent pre-treatment radiological assessment with CECT and ^18^F-FDG PET/CT for the purpose of disease stratification through the AJCC 7^th^ edition TNM staging, in order to determine the intended management plan. The CECT and ^18^F-FDG PET/CT findings were classified as true positive (positive imaging study that was confirmed histopathologically), true negative (normal imaging study with no further evidence of cancer), false positive (positive imaging study with no histopathological evidence of cancer) or false negative (normal imaging study with further proven cancer).^8^

Through ^18^F-FDG PET/CT, it was found that there were 27 true positive cases, one false positive, one false negative case and one true negative case. Three patients who were suspected of having benign lesions following a conventional clinical assessment were proven to be malignant cases after histopathological examination and ^18^F-FDG PET/CT. Two of these patients were initially diagnosed through conventional staging as having a thyroglossal cyst, but malignancy was proven through ^18^F-FDG PET/CT. This was subsequently confirmed to be papillary carcinoma, by means of histopathological examination. One of our patients initially presented with clinical and CECT features suggestive of mastoiditis and later developed widespread lesions over various sites that were positive through ^18^F-FDG PET/CT. Histopathological examination confirmed this case as metastatic adenocarcinoma. The false positive case was a patient with a parotid lesion that was positive through ^18^F-FDG PET/CT, but histopathological examination revealed this to be oncocytoma.

In our study, ^18^F-FDG PET/CT imaging accurately identified the extent of primary tumors. Thus, the tumor (T) staging changed in five patients. PET/CT imaging also correctly detected the lymph nodes and changed the node (N) staging in three patients. In this manner, ^18^F-FDG PET/CT raised the staging of 33.3% of the cases (n = 10), while 16.6% (5/30) showed changes in T-staging and metastasis (M) staging, and 10.0% (3/30) showed changes in N-staging.

The treatment plans were altered in the cases of 43.3% (13/30) of our study group patients, while there was no management change in the remaining 56.7% (17/30). 46.6% (14/30) of the patients showed stage migration, i.e., for 43.3% (13/30) the staging increased; and for 3.3% (1/30) the staging decreased. The management intent based on CECT and the changes after ^18^F-FDG PET/CT are shown in Table 3.

**Table 3.** Management intent based on CT and after PET-CT, and impact of PET-CT findings on management intent

Patient No.	Diagnosis from CT staging (pre-PET/CT)	Management intent after CT	Diagnosis after PET/CT staging	Management intent after PET/CT	Impact of PET/CT on management intent
1	Thyroglossal cyst	Excision of cyst	Papillary carcinoma T1N0M0/stage II	Thyroidectomy and radioiodine therapy	High
2	NPC T1N3M0/stage IV B	3 cycles of neoadj Ct and CtRT	NPC T1N3bM0/stage IV B	3 cycles of neoadj CT then CtRT	Low
3	Carcinoma of larynx T1aN0M0/stage I	RT	T1aN0M0/stage I	RT	Low
4	Parotid tumor	Chemotherapy	Lymphoma of parotid	Surgery	High
5	Malignant tumor of tonsils T2N0M0/stage II	Surgery	Sarcoma of tonsils T2N0M0/stage II	Surgery	Low
6	Benign cyst	Excision of cyst	Papillary carcinoma T1N1bM0/stage I	Thyroidectomy and radioiodine therapy	High
7	Benign nodule	Excision	Occult neck node carcinoma TxN1M0	Neck dissection	Low
8	Adnexal tumor TIN0M0/stage I	Excision	Adnexal carcinoma TIN0M0/stage I	Excision	Low
9	Carcinoma of larynx T1N0M0/stage I	RT	T1N0M0/stage I	RT	Low
10	NPC T1N2M0/stage III	CtRT	T1N3bM0/stage IV B	3 cycles of neoadj Ct and CtRT	High
11	NPC T2N1M0/stage II	CtRT	T4N1M0/stage IV A	3 cycles of neoadj Ct and CtRT	High
12	NPC T1N0M0/stage I	RT	T1N0M0/stage I	RT	Low
13	NPC T2N2M0/stage III	CtRT	T4N2M0/stage IV B	3 cycles of neoadj Ct and CtRT	High
14	Carcinoma of tonsils T2N3M0/stage IV B	Surgery and RT	T2N3M0/stage IV B	Surgery and RT	Low
15	Lymphoma/stage 1	Ct	Stage 3	Ct	Low
16	NPC T1N1M0/stage II	CtRT	T1N1M0/stage II	CtRT	Low
17	Mastoiditis	Surgery	Metastatic adenocarcinoma of base of skull T4N0M1	Palliative therapy	High
18	Carcinoma of oropharynx T2N1M0/stage II	Surgery and RT	T3N2M1/stage IV C	Palliative therapy	High
19	NPC T3N2M0/stage III	CtRT	T3N3M0/stage IV B	3 cycles of neoadj Ct then CtRT	High
20	NPC T3N0M0/stage III	CtRT	T4N0M1/stage IV C	Palliative therapy (6 cycles of Ct)	High
21	NPC T3N3M0/stage IV B	3 cycles of neoadj Ct then CtRT	T4N3M0/stage IV B	3 cycles of neoadj Ct then CtRT	Low
22	NPC T3N0M0/stage III	CtRT	T4N0M1/stage IV C	Palliative therapy (6 cycles of Ct)	High
23	NPC T2N2M0/stage III	CtRT	T2N2M1/stage IV C	Palliative therapy (6 cycles of Ct)	High
24	NPC T1N1M0/stage II	CtRT	T1N2M0/stage III	CtRT	Low
25	NPC T2N0M0/stage II	CtRT	T2N0M0/stage II	CtRT	Low
26	NPC T3N0M0/stage III	CtRT	T3N0M0/stage III	CtRT	Low
27	NPC T2N0M0/stage II	CtRT	T2N0M0/stage II	CtRT	Low
28	NPC T3N3M0/stage IV B	3 cycles of neoadj Ct then CtRT	T3N3M1/stage IV C	Palliative therapy (6 cycles of Ct)	High
29	Carcinoma of larynx T2N0M0/stage II	RT	T2N0M0/stage II	RT	Low
30	NPC T2N2M0/stage III	CtRT	T2N2M0/stage III	CtRT	Low

NPC = nasopharyngeal carcinoma; CT = computed tomography; PET/CT = positron emission tomography/computed tomography; RT = radiotherapy; Ct = chemotherapy; neoadj = neoadjuvant; CtRT = chemoradiotherapy.

Among the patients whose staging increased, 30% (3/10) of them benefited from addition of neoadjuvant chemotherapy. Distant metastases were identified in six patients and the management plans were changed from definitive to palliative intent. One of our patients with carcinoma oropharynx, for whom oncological surgery with radiotherapy had been planned, was found through ^18^F-FDG/PET/CT to be developing lung metastases, which was confirmed through cytological tests. The management plan was therefore changed to palliative therapy. Another patient with metastatic adenocarcinoma of the skull also had a change in the treatment plan to palliative intent. Among the remaining four patients, who had nasopharyngeal carcinoma (NPC), two presented skeletal metastases and the other two were seen to have mediastinal nodal metastases.

**Figure 1.**
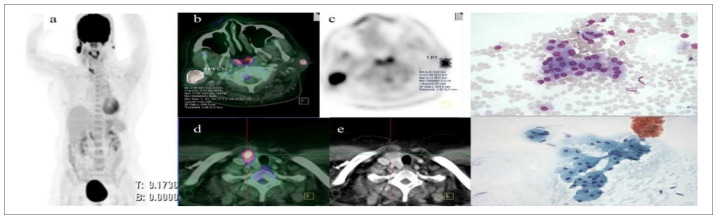
^18^F-fluorodeoxy-D-glucose positron emission tomography/computed tomography incorrectly diagnosed as malignancy of parotid in this 60-year-old woman who presented with progressively increasing parotid swelling for a duration of six months. The final diagnosis was Birt-Hogg-Dubé syndrome with benign oncocytoma of the parotid.

^18^F-FDG PET/CT decreased the staging of 6.6% of the patients (n = 2). One of these cases consisted of postoperative tonsillar sarcoma, in which there were low-activity lesions in distorted anatomy, which reflected the post-surgical change. The other case was incorrectly diagnosed as parotid lymphoma, and the final histopathological diagnosis was benign oncocytoma (Figure 1).

The influence of ^18^F-FDG PET/CT on stage migration and its impact on management intent are shown in Table 3. The clinical accuracy of ^18^F-FDG PET/CT for managing patients with head and neck cancers was derived from contingency tables.

The sensitivity, specificity, positive predictive value (PPV) and negative predictive value (NPV) of ^18^F-FDG PET/CT were 96%, 50%, 96% and 50%, respectively. The accuracy of ^18^F-FDG PET/CT for clinical evaluation of head and neck cancers was 93%. On the other hand, the sensitivity, specificity, PPV and NPV of CECT assessment were 89.2%, 50%, 96.1% and 25%. The accuracy of CECT assessment for detecting head and neck cancer was 86.7%. ^18^F-FDG PET/CT improved the sensitivity and accuracy of detection of head and neck malignancy, in comparison with CECT assessment, to 96% and 93% from 89.2% and 86.7%, respectively. The negative predictive value from the CT assessment was lower than the NPV from PET/CT imaging.

After a period of five years, we performed a search in the patients’ records at the specialist clinic. We found that 10 patients had died, 16 (53.3%) were survivors and four had been lost in the follow-up. The surviving patients had received radiotherapy alone and/or in combination with chemotherapy elsewhere and had returned. Nasopharyngeal carcinoma was the diagnosis for all the four lost patients. In the group of NPC patients (n = 17), seven patients died, while the remaining six survived. Among the dead patients, PET-CT raised the staging with regard to T-staging (n = 3), N-staging (n = 2) and M-staging (n = 3). Among the survivors, PET-CT also raised the T-staging (n = 1), N-staging (n = 3) and M-staging (n = 2). From the records of the dead patients (n = 10), seven had been diagnosed with NPC, one with lymphoma, one with metastatic adenocarcinoma at the base of the skull and one with carcinoma of the hard palate. ^18^F-FDG-PET/CT increased the T-staging in five patients, the N-staging in three patients and the M-staging in five patients, while the staging of the patient with lymphoma was increased from stage I to stage III. Among the survivors (n = 16), PET-CT changed the TNM staging of nine patients through increasing the T-staging (n = 3), N-staging (n = 4) and M staging (n = 2). There was no change in the clinical staging of the remaining seven survivors. The estimated overall mean survival after diagnosis was 43.6 months (95% confidence interval, CI = 35.2-51.9). The survival rate diminished from 86.7% during the first six months to 66.7% by the 60^th^ month of the study (Table 4).

**Table 4.** Estimated cumulative survival rate among the patients

Time (months)	Estimated cumulative survival rate
6	86.7
12	70.0
24	66.7
60	66.7

Mean survival time in months according to sociodemographic characteristics, tumor stages and treatment received was analyzed and is presented in Table 5.

**Table 5.** Mean survival time in months according to sociodemographic characteristics, tumor stages and treatment received

Factors relating to survival	Mean				P
	Estimate	Standard error	95% confidence interval		
			Lower bound	Upper bound	
**Race**					
Malay	48.500	5.851	37.033	51.455	0.09 0.327
Chinese	42.000	5.927	30.383	59.937	
Indian	12.000	0.000	12.000	12.000	
Overall	43.600	4.270	35.230	51.970	
**Gender**					
Male	44.000	5.395	33.425	54.575	0.947
Female	43.000	6.958	29.362	56.638	
Overall	43.600	4.270	35.230	51.970	
**Age group**					
Less than 65 years	46.615	4.333	38.122	55.109	0.026
Greater than or equal to 65	11.023	11.023	2.396	45.604	
	43.000	4.270	35.230	51.970	
**Tumor stage**					
T0_T2	51.000	8.216	34.897	67.103	0.365
T3_T4	40.957	5.000	31.157	50.756	
**Treatment**					
Curative	50.250	3.918	42.571	57.929	0.001
Palliative	17.000	7.927	1.464	32.536	
Overall	43.600	4.270	35.230	51.970	

For all of our patients with head and neck malignancies, the Kaplan-Meier estimate for mean survival time (with standard error) for those age less than 65 years old was 46.6 (4.3) [95% CI = 38.1-55.1]; while for those aged 65 years and over, the estimate was 24.0 (11.0) [95% CI = 2.4-45.6]. The log-rank test revealed a statistically significant difference between the survival rates over time (P = 0.026). It was found that the mean survival time (with standard error) of the patients who received definitive treatment (surgery alone, surgery with radiotherapy, radiotherapy alone and radiotherapy with chemotherapy) was 50.3 (3.9) [95% CI = 42.6-57.9]. On the other hand, those who received palliative treatment had mean survival (with standard error) of 17.0 (7.9) [95% CI = 1.5-32.5]. This difference was statistically significant, with a P-value of 0.001.

## DISCUSSION

Head and neck cancers encompass a heterogeneous group of tumors that are a biologically aggressive and therapeutically challenging category of disease.^7-9^ The appropriate management decision for this complex form of cancer is based on the primary site, histological subtype, stage, resectability, patient’s fitness and treatment preference.^10^ Accurate staging is crucial for selection of the appropriate treatment modality in individual patients.

CT and MRI are widely used as the first-line imaging approach for staging of head and neck cancer. Both of these imaging modalities rely on morphological criteria like size and contrast enhancement patterns, which are not particularly specific for detection of metastases.^9,11 18^F-FDG PET/CT has been shown to yield promising results for diagnosing and staging of head and neck squamous cell carcinoma, compared with standard imaging modalities.^12^

In our study, we sought to prospectively evaluate the influence of ^18^F-FDG PET/CT on the initial staging and its impact on the treatment plan for head and neck cancer patients. The data for this study consisted of information from our own patients, in contrast with the data in other, multicenter studies, which relied on medical records. The majority of our study patients (56.7%) were diagnosed with nasopharyngeal carcinoma, which was in accordance with the epidemiological pattern of head and neck cancers in Malaysia. Nasopharyngeal carcinoma is one of the ten most common cancers in this multiracial Southeast Asian country.

Our results demonstrated that ^18^F-FDG PET/CT significantly changed the overall multidisciplinary team decision regarding treatment intent, compared with clinical conventional CT staging. These changes to staging caused significant reclassification of patients’ treatment decisions and their overall survival prognosis. The impact of ^18^F-FDG PET/CT on the treatment decision was mainly due to the improvement in the accuracy of staging.

The data from our study demonstrated that ^18^F-FDG PET/CT raised the staging in the cases of 33.3% (n = 10) of the patients. These data were in accordance with the findings from various published studies, which demonstrated management changes in the cases of 31-34% of head and neck cancer patients.^8,13,14,15^ Meanwhile, the T-staging in our study cohort was changed in 16.6% (5/30) of the patients. In a study by Antoch et al., the T-staging was accurately determined in 82% of the cases, through use of fused PET/CT.^16^ PET/CT can reveal the full tumor extent, even when a tumor is ill-defined with submucosal extent and diffuse infiltration. A study by Tantiwongkosi et al. showed that PET/CT could be helpful in identifying subtle but focally hypermetabolic NPC, when the CT and MRI findings are not obvious.^17^

Precise detection of cervical lymph node metastases is crucial for planning the surgical margins and radiotherapy.^18^ According to our study, the N-staging was changed in 10% of the cases. A meta-analysis by Sun et al. showed that ^18^F-FDG PET/CT had good diagnostic performance, compared with conventional imaging, for detection of regional nodal metastases.^19^

Our study supports the notion that ^18^F-FDG PET/CT is effective in detecting distant metastases. Notably, 26.6% of the study patients had metastases and modification of M-staging. This modification was due to the higher sensitivity of ^18^F-FDG PET/CT for detecting certain subtle lesions, which can be missed through conventional imaging or a single-stop modality with whole-body coverage.

PET/CT accurately detected skeletal metastases in two of our NPC patients. Three patients of our study group were found through PET/CT to be developing mediastinal nodal metastases. Another patient was seen to have widespread metastases in various organs. The treatment for all these patients with distant metastases was subsequently revised to palliative intent. Head and neck cancer patients with distant metastases are not considered curable, and most cases lead to palliative treatment strategies.^20^ Therefore, detection of distant metastases is important because this avoids unnecessary or inappropriate treatment. ^18^F-FDG PET/CT imaging may prevent unnecessary surgery in some patients, in whom this would have been associated with high morbidity and functional impairment, through identifying locoregional and distant metastases.^21^

The treatment plans were changed in 43.3% (13/30) of our patients, while no management change was made in the cases of the remaining 56.7%. Our study results showed changes that were similar to what was observed by Veit-Haibach et al.^22^ In that study, the accuracies of TNM staging using PET/CT and CT were compared, and it was found that staging based on PET/CT imaging changed the therapy for 42% (13/31) of the patients, compared with therapy based only on CT.^22^ In another study by El-Khodary et al., treatment changes were made in the cases of 41.7% of the patients.^9^

A variety of changes to treatment were made among our patients. These included addition of chemotherapy or radiotherapy and abandonment of localized surgery and radiotherapy with curative intent, which was replaced by treatment with palliative intent. The aim of chemotherapy was shifted from curative to palliative intent in 20% (6/30) of our patients. These patients were in a group at an advanced stage with presence of distant metastases. Our data were found to be consistent with the findings of previously published studies.^13,23-25^

In the present study, ^18^F-FDG PET/CT was found to have improved sensitivity and accuracy for detecting head and neck malignancy, in comparison to conventional CECT. The sensitivity, specificity, PPV, NPV and accuracy of ^18^F-FDG PET/CT were reported to be 96%, 50%, 96%, 50% and 93% respectively. This was comparable to the study published by Gordin et al in 2007.^26^

During the follow-up of our study group patients, we found that 10 patients had passed away. These patients’ treatments were therefore reclassified from having curative to having palliative intent. This notably strengthens the argument that PET/CT has a major incremental impact with regard to identifying high-risk patients who do not benefit from aggressive curative treatment.

In interpreting ^18^F-FDG PET/CT imaging, the challenges include physiological uptake of fluorodeoxy-D-glucose (FDG) by normal tissues, false positive results due to inflammation, limited resolution of small lesions and motion artefacts.^27,28^ Cost-effectiveness is the major consideration in deciding whether to use of ^18^F-FDG PET/CT as part of the initial imaging. Its cost needs to be weighed against the benefit of early detection of distant metastases, synchronous primary and resulting interventions.^29^

Our study had several limitations. The majority of our study cohort were NPC patients, which might have introduced a working bias. Moreover, our sample consisted of a small number of patients with head and neck cancers at different sites. Because of these limiting factors, the results from our study focused mainly on nasopharyngeal carcinoma and may not have reflected the situation regarding other head and neck cancers. Further prospective studies comprising larger patient cohorts are required in order to ascertain the impact of ^18^F-FDG PET/CT on the management of various head and neck malignancies.

## CONCLUSION

In conclusion, our study demonstrated that ^18^F-FDG PET/CT had higher sensitivity and accuracy for detecting head and neck malignancy than those of conventional CECT. ^18^F-FDG PET/CT provides additional information and accurate staging, which assist in planning for adequate treatment and in minimizing treatment-related toxicity and functional impairment. From our study findings, we would advocate for incorporation of ^18^F-FDG PET/CT into the initial staging of clinically advanced head and neck malignancy.
